# Changing relative and absolute socioeconomic health inequalities in Ontario, Canada: A population-based cohort study of adult premature mortality, 1992 to 2017

**DOI:** 10.1371/journal.pone.0230684

**Published:** 2020-04-02

**Authors:** Emmalin Buajitti, John Frank, Tristan Watson, Kathy Kornas, Laura C. Rosella

**Affiliations:** 1 Dalla Lana School of Public Health, University of Toronto, Toronto, Ontario, Canada; 2 ICES, Toronto, Ontario, Canada; 3 The Usher Institute of Population Health Sciences and Informatics, College of Medicine and Veterinary Medicine, University of Edinburgh, Edinburgh, United Kingdom; University of California-Irvine, UNITED STATES

## Abstract

**Background:**

This study aimed to characterize trends in absolute and relative socioeconomic inequalities in adult premature mortality between 1992 and 2017, in the context of declining population-wide mortality rates. We conducted a population-based cohort study of all adult premature deaths in Ontario, Canada using provincial vital statistics data linked to census-based, area-level deprivation indices for socioeconomic status.

**Methods:**

The cohort included all individuals eligible for Ontario’s single-payer health insurance system at any time between January 1, 1992 and December 31, 2017 with a recorded Ontario place of residence and valid socioeconomic status information (N = 820,370). Deaths between ages 18 and 74 were used to calculate adult premature mortality rates per 1000, stratified by provincial quintile of material deprivation. Relative inequalities were measured using Relative Index of Inequality (RII) measures. Absolute inequalities were estimated using Slope Index of Inequality (SII) measures. All outcome measures were calculated as sex-specific, annual measures for each year from 1992 to 2017.

**Results:**

Premature mortality rates declined in all socioeconomic groups between 1992 and 2017. Relative inequalities in premature mortality increased over the same period. Absolute inequalities were mostly stable between 1992 and 2007, but increased dramatically between 2008 and 2017, with larger increases to absolute inequalities seen in females than in males.

**Conclusions:**

As in other developed countries, long-term downward trends in all-cause premature mortality in Ontario, Canada have shifted to a plateau pattern in recent years, especially in lower- socioeconomic status subpopulations. Determinants of this may differ by setting. Regular monitoring of mortality by socioeconomic status is the only way that this phenomenon can be detected sensitively and early, for public health attention and possible corrective action.

## Introduction

Socioeconomic disparities–specifically, an association between low socioeconomic status and poorer outcomes and experiences of health–are well established for indicators of disease status [1, 2], quality of health care [3, 4], and other risk factors for poor health [5, 6]. Tracking these inequalities has been adopted as a core dimension of population and public health surveillance efforts [7]. More recently, there has also been interest in understanding health inequalities as a barometer of underlying societal problems; that is, that socioeconomic disparities in health may widen in response to damaging social or political change [8]. Ongoing monitoring of health inequalities, particularly in terms of how they change over time, thus offers considerable insight regarding the health and social conditions of a given population.

Relative measures of socioeconomic inequalities have repeatedly shown a widening mortality gap between low- and high-socioeconomic status groups in the United States [9, 10], Canada [11], and many European countries [12, 13] since at least the 1990s. Additional concern has recently arisen regarding population-level mortality rates in the US and UK, which appear to have increased this decade after decades of continuous and steady decline [14, 15]. In the United States, rising mortality is hypothesized to be related to increasing ‘deaths of despair’ in white middle-aged groups, particularly those of low educational attainment [16]. In the United Kingdom, an increase in deaths among the elderly may be driven by increased austerity and reductions to old-age social care [17]. These phenomena are not yet well understood but suggest a complex interplay between population health and underlying social conditions. Even without a full explanation, it appears we may be in a time of change for health status at the population level.

More evidence is needed to clarify how mortality trends in Canada compare to those in other countries. Relative inequality measures for the Ontario and Canadian populations suggest that, as reported in other countries, socioeconomic inequalities in mortality and premature mortality have risen in recent years [11, 18]. Possibly as a result of population aging, crude mortality rates in Canada and Ontario rose between 2013 and 2016; premature mortality, which captures only deaths before age 75, was neither declining nor increasing in Ontario as of 2015 [19, 20]. However, no published work has studied both long-term and recent trends in socioeconomic inequalities, along with trends in population-level rates.

The present study, of all adult premature mortality in Ontario, aims to provide this context. Ontario is the largest province in Canada, with 38.6% of the Canadian population in 2017 [21], and has the largest multi-linked repository of administrative health data in the country, at ICES [22]. We analysed 26 years of population vital statistics data to understand how the association between socioeconomic status and premature mortality has changed in Ontario, with two primary objectives: [1] to describe the relationship between socioeconomic inequities and population health between 1992 and 2017; and [2] to understand Ontario’s place in the growing body of literature characterizing recent, regressive trends in population mortality.

## Materials and methods

### Data sources

The analysis used population-based vital statistics data from the Ontario Registrar General’s death file (ORG-D). ORG-D was linked via deterministic and probabilistic linkage to demographic information from the Registered Persons Database, a central population registry based on eligibility for Ontario’s single-payer health insurance plan. This linkage has been described elsewhere [22]. Between January 1, 1992 and December 31, 2017, 820 370 premature deaths were registered in ORG-D and successfully linked, with a success rate of greater than 95% overall and greater than 98% for each year after 2003.

### Primary outcome

Adult premature mortality was defined to include all deaths between the ages of 18 and 74 registered in Ontario between 1992 and 2017, with a valid Ontario residence at death. These ages align with the definition of premature mortality used by the Canadian Institute for Health Information [23]. They are also consistent with prior analyses of premature mortality that have been carried out in Ontario [18, 24] and other similar jurisdictions [25–27].

### Socioeconomic status

Assessment of socioeconomic status used data from the Ontario Marginalization Index (ON-Marg)[28]. ON-Marg, which was developed using iterative factor analysis of area-level Census data, is an index of four domains of sociodemographic characteristics—residential instability, material deprivation, dependency, and ethnic concentration [29]. Factor scores for each domain are generated for several levels of geography within Ontario, the smallest of which is the dissemination area (DA) with a population of between 400 and 700. We used the DA-level factor scores for material deprivation to capture socioeconomic status in our study cohort. The calculation of material deprivation factor scores includes variables for traditional socioeconomic status indicators such as income, education, and employment, and has been previously shown as the ON-Marg domain most strongly associated with health outcomes [30].

ON-Marg scores have been produced for all Canadian census years since 2001: 2001, 2006, 2011 and 2016. Using nearest-census ON-Marg data, each death record in our cohort was assigned to a provincial quintile of material deprivation according to their RPDB postal code at death, using Statistics Canada’s Postal Code^OM^ Conversion File Plus [31]. Records with missing postal code information, non-Ontario residents, and deaths which could not be linked to ON-Marg data were excluded from the analysis. The total number of deaths excluded was 32 130 or 3.8%.

Although we used an area-level deprivation index to assign socioeconomic status in our cohort, all analyses were carried out on individual person-level records (i.e. not the aggregate units). Our findings should thus be interpreted as a descriptive summary of socioeconomic inequalities in the Ontario population, where socioeconomic status is represented by the material deprivation of the area in which an individual resides at time of death. Area-level measures of socioeconomic status provide important information about a population that is not captured by individual-level income measurement, such as: area-level infrastructure, access to key health-promoting services, and environmental exposures [32]. However, our findings may not be representative of the socioeconomic trends that would be seen if measured at the individual level [33].

### Statistical analyses

Two important methodological considerations informed our analytic approach. First, we wished to present parallel measures for absolute and relative measures of inequality. Reporting both absolute and relative measures is recommended by the STROBE reporting guidelines for observational studies [34]. In studies of health inequalities specifically, using both absolute and relative measures is considered a more balanced and appropriate choice to fully contextualize the relationship between socioeconomic position and health [35, 36]. We chose to summarize relative and absolute inequalities using relative index of inequality (RII) and slope index of inequality (SII), respectively. Second, we wanted to consider premature mortality risk, and socioeconomic inequalities therein, simultaneously. There is an established mathematical relationship between measures of inequality and underlying population rates, such that they tend to vary together in predictable ways; for example, relative inequalities in mortality rates have been shown to increase in many countries as overall mortality declines [37]. For interpretational clarity, we wished to visualize premature mortality rates, absolute inequalities in premature mortality, and relative inequalities in premature mortality collectively. We adopted a typology proposed by Blakely et al. [38] which presents a comprehensive approach to the study of mortality and inequalities over time.

Adult premature mortality rates were calculated as deaths per 1000, by sex and quintile of material deprivation, for each year in the study period. Age-specific rates were calculated for the following age groups: 18–34, 35–44, 45–54, 55–64 and 65–74. The mid-year Ontario population for ages 18 to 74 was used as the denominator for rate calculations.

Annual RII and SII measures were estimated using unadjusted Cox proportional hazards models and additive hazards models, respectively, as proposed by Moreno-Betancur et al.[39]. The independent variable was approximated socioeconomic rank, set as the proportion of the population of lower socioeconomic position than the midpoint of a given quintile (i.e. for quintile 1, rank = 0.1; quintile 2, rank = 0.3; up to quintile 5, rank = 0.9). No additional covariates were included in the model. For both SII and RII, the health outcome was the hazard rate of premature mortality, which for the additive SII model was linearly associated with the exposure; the Cox model, which was used to estimate RII, specifies a log-linear relationship between socioeconomic rank and premature mortality hazard rate.

This approach builds from the definition of RII proposed by Mackenbach and Kunst[36], where RII represents the risk *ratio* of the hypothetically worst-off person in a population (i.e., 0^th^ percentile of socioeconomic status) compared to the hypothetically best-off person in the same population (100^th^ percentile), and SII represents the risk *difference* between the same groups. Thus, an RII of 2 would be interpreted to mean that the extreme lowest socioeconomic position in a population is associated with twice the risk of premature mortality of the extreme highest socioeconomic position in that same population. An SII of 2 per 1000 would be interpreted to mean that the mortality risk in the extreme lowest socioeconomic position is 2 deaths per 1000 greater than the mortality risk in the extreme highest socioeconomic position, again within the same population.

There are several available measures for quantifying absolute and relative inequalities. We chose to report RII and SII for the following reasons. Firstly, it is a model-generated estimate that considers information from across the socioeconomic gradient in the population, which is not case for simpler measures. Secondly, because it is analogous to relative risk and risk difference measures, it has a straightforward interpretation to those who use these measures, which are common in public health and epidemiology. Lastly, RII and SII are summary measures that represent overall socioeconomic inequality in the population as a single term, which allows direct comparisons over time and between groups.

Some approaches to estimating RII and SII assume a linear association between socioeconomic status and health outcomes across the population. In order to avoid this assumption, we utilized the approach given by Moreno-Betancur et al., which estimates SII and RII without assuming a true linear association in the underlying data [39]. RII and SII were thus estimated using sex-specific models for the association between socioeconomic rank and hazard of premature mortality. An additive model was used to estimate SII, which were measured in deaths per 1000 to align with our prior rate calculations. Both RII and SII calculations were carried out separately for males and females, and for each year between 1992 and 2017.

### Visualization of trends

To visually represent our three outcome measures (mortality rates, RII and SII) simultaneously, we used the data visualization approach proposed by Blakely et al.[38]. These graphics place the overall (crude) population premature mortality rate on the x-axis, with RII on the y-axis. Points are connected in order of year, with arrows indicating the directionality of the trend. As visualized in these plots, SII values are a transformed representation of the estimated RII values, based on the following mathematical relationship between rate, RII and SII: SII = 2 × rate × (RII– 1) ÷ (RII + 1) [7]. This equation is based on the Mackenbach and Kunst conceptualization of RII and SII, and thus represents a linearized approximation of SII[38]. Our model-generated estimates of SII, which should be considered more robust compared to the transformed RII values (contour lines), are presented separately in tables.

### Age-adjusted analyses

As they are age-conditional, we do not further standardize premature mortality rates for age. Though age structure may differ between socioeconomic groups (even within the restricted 18 to 74 population), we believe that age-standardizing after conditioning on age may obscure true premature mortality trends[40]. However, to ensure transparency as to whether using age-standardized rates would meaningfully impact our findings, we repeated our analysis accounting for potential age differences in the following ways: age-standardizing the premature mortality rates to the 2000 Canadian standard population, using age as the time scale (in place of time in study) for RII calculations, and age-standardizing the SII calculations.

### Ethics statement

This study was approved by the University of Toronto Health Sciences Research Ethics Board (Protocol 32405). ICES is a prescribed entity under section 45 of Ontario’s Personal Health Information Protection Act. The use of data in this project was authorized under section 45 and did not require informed consent. All data were de-identified prior to access.

## Results

### Cohort characteristics

Socioeconomic characteristics of the cohort are described in Table 1. Adult premature deaths registered in Ontario between 1992 and 2017 occurred more frequently in males than in females. A larger proportion of deaths took place among low socioeconomic status groups (high material deprivation). This socioeconomic gradient was observed in all ages, with a particularly strong trend seen in the younger age groups (18–34 and 35–44).

**Table 1:** Sociodemographic characteristics of cohort at date of death.

		Q1 (least deprived)		Q2		Q3		Q4		Q5 (most deprived)	
		n	%	n	%	n	%	N	%	n	%
Sex	M	71611	14.47	86285	17.44	97589	19.72	109105	22.05	130196	26.31
** **	F	48745	14.97	56493	17.35	63572	19.53	71184	21.86	85590	26.29
Age	18–34	5510	14.95	6391	17.33	7066	19.17	7844	21.28	10057	27.28
	35–44	7353	14.68	8334	16.64	9445	18.85	10828	21.61	14139	28.22
	45–54	16980	14.85	19922	17.42	21629	18.91	24519	21.44	31324	27.39
	55–64	32547	14.68	38029	17.15	43570	19.65	48250	21.76	59295	26.75
	65–74	57966	14.59	70102	17.64	79451	20.00	88848	22.36	100971	25.41

### Premature mortality rates

**Fig 1:**
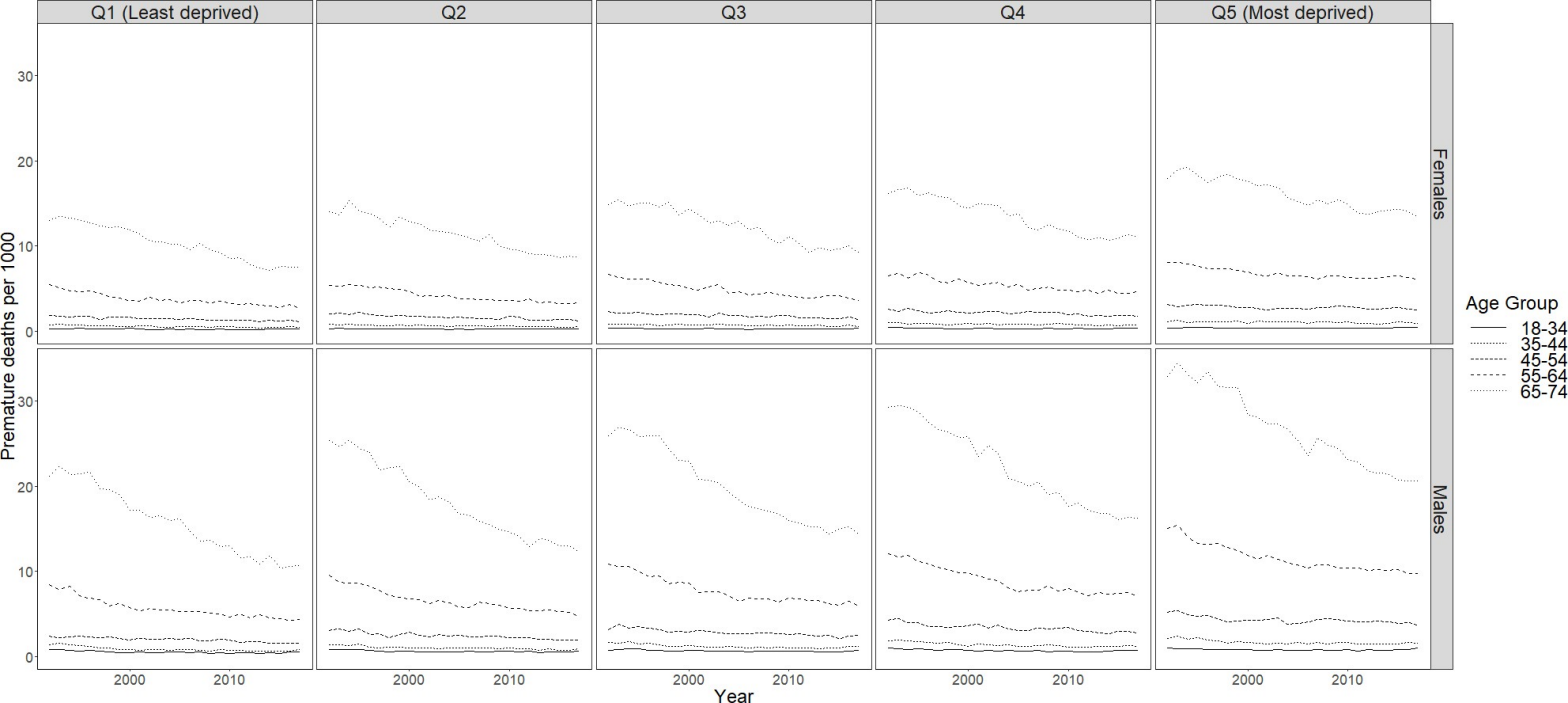
Premature mortality rates by age group, sex and material deprivation quintile, Ontario, 1992 to 2017.

Overall Ontario rates by sex for all material deprivation quintiles combined are in Table 2. Between 1992 and 2017, large decreases in premature mortality were achieved in Ontario for males and females. For males, adult premature mortality rates declined by 24.2 percent, with a corresponding 18.3 percent decline seen in females (Table 2). Improvements between 1992 and 2017 were observed for all quintiles of socioeconomic status (Fig 1).

**Table 2:** Relative and absolute inequalities in adult premature mortality (ages 18 to 74), 1992 to 2017, Ontario.

	MALES			FEMALES		
Year	MR^1^ (per 1000)	RII^2^ (95% CI)	SII^3^ per 1000 (95% CI)	MR^1^ (per 1000)	RII^2^ (95% CI)	SII^3^ per 1000 (95% CI)
**1992**	5.20	1.78 (1.69, 1.88)	3.79 (3.38, 4.21)	3.17	1.75 (1.64, 1.87)	1.79 (1.48, 2.10)
**1993**	5.32	1.82 (1.73, 1.91)	4.11 (3.66, 4.57)	3.23	1.82 (1.71, 1.94)	1.77 (1.46, 2.08)
**1994**	5.23	1.71 (1.62, 1.79)	3.56 (3.16, 3.96)	3.23	1.76 (1.65, 1.88)	1.66 (1.38, 1.94)
**1995**	5.14	1.68 (1.59, 1.76)	3.36 (2.99, 3.74)	3.21	1.77 (1.66, 1.89)	1.77 (1.48, 2.05)
**1996**	5.02	1.76 (1.67, 1.85)	3.60 (3.21, 4.00)	3.13	1.72 (1.61, 1.84)	1.57 (1.30, 1.83)
**1997**	4.81	1.82 (1.73, 1.91)	3.69 (3.30, 4.08)	3.02	1.87 (1.75, 2.00)	1.93 (1.65, 2.2)
**1998**	4.65	1.83 (1.74, 1.93)	3.69 (3.35, 4.03)	2.98	1.89 (1.77, 2.02)	1.94 (1.68, 2.21)
**1999**	4.59	1.92 (1.83, 2.03)	3.81 (3.45, 4.18)	2.93	1.82 (1.71, 1.94)	1.79 (1.54, 2.05)
**2000**	4.40	1.90 (1.81, 2.01)	3.62 (3.27, 3.97)	2.86	1.81 (1.70, 1.93)	1.76 (1.51, 2.00)
**2001**	4.23	1.91 (1.81, 2.01)	3.52 (3.18, 3.86)	2.79	1.92 (1.80, 2.04)	1.79 (1.53, 2.04)
**2002**	4.18	1.99 (1.89, 2.10)	3.59 (3.25, 3.93)	2.75	2.00 (1.87, 2.13)	1.93 (1.68, 2.18)
**2003**	4.20	2 .00 (1.90, 2.10)	3.61 (3.26, 3.96)	2.76	2.04 (1.91, 2.17)	1.97 (1.72, 2.22)
**2004**	4.06	1.88 (1.79, 1.98)	3.18 (2.86, 3.50)	2.64	1.92 (1.80, 2.04)	1.81 (1.56, 2.06)
**2005**	3.94	1.87 (1.77, 1.97)	3.15 (2.82, 3.48)	2.65	1.99 (1.87, 2.12)	1.60 (1.37, 1.84)
**2006**	3.88	1.92 (1.83, 2.03)	3.12 (2.80, 3.44)	2.55	1.85 (1.74, 1.97)	1.60 (1.37, 1.84)
**2007**	4.00	2.18 (2.07, 2.30)	3.77 (3.44, 4.11)	2.61	1.97 (1.85, 2.10)	1.77 (1.52, 2.03)
**2008**	3.96	2.20 (2.08, 2.31)	3.73 (3.40, 4.05)	2.61	2.08 (1.96, 2.22)	2.10 (1.87, 2.33)
**2009**	3.90	2.05 (1.94, 2.16)	3.88 (3.55, 4.21)	2.57	2.02 (1.89, 2.15)	2.25 (2.00, 2.50)
**2010**	3.86	2.02 (1.91, 2.13)	3.81 (3.48, 4.14)	2.55	2.00 (1.88, 2.14)	2.17 (1.93, 2.41)
**2011**	3.82	2.12 (2.01, 2.24)	4.00 (3.65, 4.34)	2.49	1.93 (1.81, 2.06)	2.01 (1.76, 2.25)
**2012**	3.80	2.15 (2.04, 2.26)	3.84 (3.51, 4.17)	2.49	2.04 (1.92, 2.17)	2.06 (1.81, 2.31)
**2013**	3.83	2.19 (2.08, 2.30)	3.98 (3.61, 4.35)	2.49	2.17 (2.04, 2.31)	2.40 (2.15, 2.65)
**2014**	3.89	2.18 (2.08, 2.30)	3.96 (3.61, 4.32)	2.56	2.34 (2.21, 2.49)	2.54 (2.26, 2.81)
**2015**	3.85	2.37 (2.25, 2.49)	4.26 (3.91, 4.61)	2.61	2.41 (2.27, 2.57)	2.65 (2.39, 2.90)
**2016**	3.97	2.32 (2.21, 2.43)	4.33 (3.98, 4.67)	2.67	2.22 (2.10, 2.36)	2.52 (2.26, 2.78)
**2017**	3.94	2.36 (2.25, 2.48)	4.10 (3.75, 4.45)	2.59	2.28 (2.15, 2.42)	2.56 (2.30, 2.81)
**% change**	-24.2	32.6	8.2	-18.3	30.3	43.0

^1^Adult premature mortality rates (deaths ages 18–74 per 1000).

^2^Relative Index of Inequality (RII) hazard ratio estimates, generated from unadjusted Cox proportional hazards models.

^3^Slope Index of Inequality (SII) hazard ratio estimates per 1000, generated from unadjusted additive hazards models.

Adult premature mortality rates by sex, material deprivation quintile and age group are shown in Fig 1A downward year-over-year trend in adult premature mortality was consistently seen for males and females between 1992 and 2006 (Fig 1). This downward trend was most strongly observed among the older age groups (55–64 and 65–74). After 2006, improvements appear to have stalled in the most-deprived group for both sexes, with notable increases in premature mortality rates seen in low socioeconomic status females for some age groups. For other socioeconomic quintiles and age groups, adult premature mortality rates were static or showed minor improvements for this period.

### Relative and absolute inequalities

Model-generated RII and SII estimates (hazard ratios) are shown in Table 2. Relative inequalities, as measured by RII, increased steadily between 1992 and 2017 (32.6 increase in males, 1992 to 2017; 30.3% in females). The magnitude of the observed relative inequalities was consistently similar for males and females, with minimal differences in RII estimates seen between sexes (Table 2). Absolute inequalities, as measured by SII, showed no substantial trend between 1992 and 2006, then increased from 2007 to 2017. Furthermore, the increase in SII was noticeably greater in females than in males (an increase of 43% compared to 8.2%).

**Fig 2:**
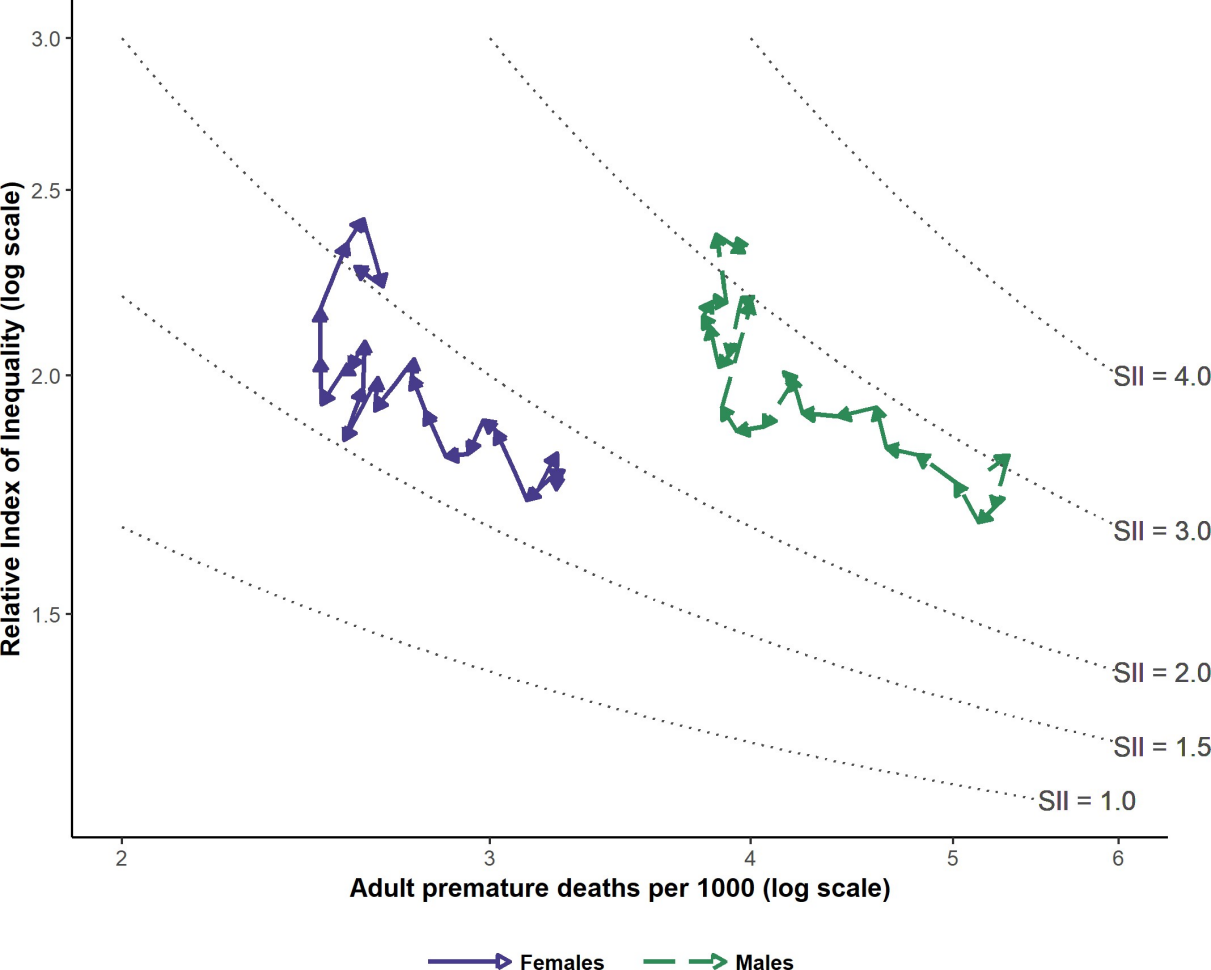
Absolute and relative inequalities in adult premature mortality (deaths ages 18 to 74), Ontario by sex, 1992 to 2017.

Trends in adult premature mortality rates, relative inequalities and absolute inequalities are visualized together in Fig 2. As described earlier, this figure plots adult premature mortality rates (x axis) versus RII (y axis), with arrows and calendar-year labels indicating the time interval from year to year. Each path–one each for males and females–thus represents the overall trend through the study period for the association between relative inequality in premature mortality by socioeconomic status and adult premature mortality. The figure shows that between 1992 and 2017, adult premature mortality rates steadily decreased (decreasing values along x axis) while relative inequalities steadily increased (increasing values along y). For much of the study period, absolute inequalities remained fairly static; here the paths move in parallel to the SII contour lines. Approaching 2017, there is a demonstrable increase in absolute inequalities, above and beyond what is expected from the simple algebraic relationship between overall rate and SII, as the trend-line paths diverge vertically upwards from the SII contours. This period, which represents approximately 2007 to 2017, represents an era of rapidly increasing relative inequalities, increasing absolute inequalities, and vanishing declines in overall premature mortality rates–especially in low socioeconomic status females.

### Age-adjusted analyses

The results of supplementary age-adjusted analyses are shown in S1 File. The direction of observed trends after age adjustment were similar to those seen in our primary analysis. However, the magnitude of change appeared larger after age adjustment, reflected by larger declines in premature mortality (-42% adjusted vs. -24% unadjusted in males; -36% adjusted vs. -18% unadjusted in females) and larger increases in RII (41% vs. 33% in males; 49% vs. 30% in females). Also, the increase in absolute inequalities in premature mortality was no longer observed for males after age-standardizing SII measures. These findings are expected, given that low socioeconomic groups in Ontario are younger than high socioeconomic status groups (see Table 1).

## Discussion

### Discussion of main findings

Recent studies in the US, UK, France, Spain, Denmark, and Switzerland[16, 17, 41, 42] have also–like this study in Ontario–shown a marked deceleration in the long-term downward trend of adult premature mortality at the entire population level, typically most pronounced among the most socioeconomically disadvantaged. In Ontario’s case, we have shown that the subpopulation at greatest risk is the lowest- socioeconomic status quintile in both sexes, with low socioeconomic status women especially affected. In the USA, this reversal of previous long-term adult mortality trends was first evident some years earlier, and largely restricted to non-Hispanic whites, at a wider range of ages; it also especially affected persons with a low level of completed education[16, 41]. In the UK, on the other hand, this plateauing of long-term adult mortality declines manifested somewhat later (typically after 2014), and has been more marked in older (age 75+) rather than middle-aged adults, again especially in females[17].

Whatever the precise cause of these new trends in premature adult mortality, their concentration among the more deprived segments of the population marks a new era in population health surveillance. Like the proverbial “canaries in the mine” of yesteryear–which were place in mines to alert workers early to worsening of air quality–the regular monitoring of mortality trends by socioeconomic status has thus been vindicated, in terms of its public health utility. Without such sensitive monitoring of mortality inequalities, it seems likely that the detection of such new trends would have been delayed for at least a few years, simply because their concentration in deprived socioeconomic status groups leads to their dilution at the whole-population level.

This paper also demonstrates the benefits of using newer methods of modelling and depicting the relationship between overall population mortality rate trends and the importance of monitoring absolute and relative inequalities in the context of falling premature mortality rates. These methods help to detect as early as possible when annually computed SIIs, a major summary measure of health inequalities, in widespread use, which are algebraically composed to ensure their rise as overall mortality rates fall–become sufficiently “off-trajectory” to raise statistically legitimate concern.

### Limitations

Consistent attribution of socioeconomic status over a 26-year period is difficult, and sensitive to our choice of socioeconomic measure. ON-Marg, which was calculated for 2001, 2006, 2011 and 2016 Canadian census cycles, is further influenced by the availability of robust population socioeconomic information[29]. We chose to exclude ON-Marg data from 2011, when the development of the index was altered significantly to account for the replacement of the mandatory long-form census with the 2011 National Household Survey. Additionally, we carried out a sensitivity analysis estimating all outcome measures using area-level income quintiles, which had no meaningful impact on observed trends.

We used quintile measures, which are a measure of socioeconomic status relative to the entire population. As a result, the reported RII and SII estimates describe socioeconomic inequalities in premature mortality, for the Ontario population in a given year. However, these measures may be influenced by changes over time to the Ontario population–for example, the settlement of a large number of immigrants, who have a well-described health advantage in Ontario [18]. There may also have been intra-provincial changes in socioeconomic distribution, such as displacement of low-income residents from gentrifying neighbourhoods, which may have influenced the socioeconomic inequalities indicated by RII and SII measures. In the context of the current study, we cannot separate these population-level sociodemographic changes from the socioeconomic conditions of the neighbourhoods themselves.

Because Ontario administrative health data do not capture individual-level socioeconomic data (i.e. income) for the entire population, our analysis was reliant on area-level measures of socioeconomic status. While both area- and individual-level socioeconomic status measures are associated with health-related characteristics, concordance between measures has been shown to be poor in the Ontario population[43]. Thus, the health inequalities described in this paper do not directly reflect associations between individual-level deprivation and mortality. In the future, it would be of great value to Canadian population health research if alternative individual- or household-level measures of socioeconomic status could be integrated either by linking existing socioeconomic data sources (e.g. administrative tax data [44]) or creating novel indices of socioeconomic status (such as the HOUSES Index of housing characteristics [45]).

The present study does not provide evidence of a causal relationship between area-level socioeconomic status and premature mortality. Many factors, including health status, may determine an individual’s neighbourhood of residence. Additionally, we did not account for residential movement; that is, decedents’ area-level socioeconomic status at death may not be representative of exposure through their life course.

### Potential implications

Many countries around the world are now embarking in earnest efforts to measure and monitor health inequalities by socioeconomic status, in part to tackle Sustainable Development Goal #10: “Reduce health inequalities within and between countries”[46–48]. In many low- and middle-income countries, measuring such inequalities–including those in mortality–is fraught by the absence of reliable and complete vital statistics registration, poor-quality census data, and a lack of any accurate system to characterize the socioeconomic profile, either of individuals, or of local-small-areas of residence[49]. The present study provides an additional rationale for countries developing routinely usable and relatively inexpensive means of monitoring inequalities in mortality by socioeconomic status: without such monitoring systems, significant decelerations in long-term downward trends in mortality are unlikely to be detected early, and thus acted upon by public health authorities in a timely manner.

## Supporting information

S1 File Age-adjusted analyses. (PDF)
