# Supplementary material for: Changing relative and absolute socioeconomic health inequalities in Ontario, Canada: A population-based cohort study of adult premature mortality, 1992 to 2017
Source: PLoS One. 2020 Apr 2;15(4):e0230684. doi: 10.1371/journal.pone.0230684 (PMC7117737; doi:10.1371/journal.pone.0230684)

S1 Table. Age-adjusted relative and absolute inequalities in adult premature mortality (ages 18 to 74), 1992 to 2015, Ontario.

	MALES			FEMALES		
**Year**	**MR (per 1000)**	**RII (95% CI)**	**SII (95% CI)**	**MR (per 1000)**	**RII (95% CI)**	**SII (95% CI)**
**1992**	5.45	1.80 (1.71, 1.90)	3.00 (2.71, 3.34)	3.29	1.56 (1.46, 1.67)	1.39 (1.13, 1.59)
**1993**	5.49	1.87 (1.77, 1.97)	3.34 (3.10, 3.69)	3.29	1.64 (1.53, 1.75)	1.53 (1.18, 1.67)
**1994**	5.33	1.77 (1.68, 1.86)	2.82 (2.57, 3.1)	3.26	1.60 (1.50, 1.71)	1.47 (1.23, 1.68)
**1995**	5.20	1.76 (1.67, 1.85)	2.60 (2.40, 2.99)	3.22	1.64 (1.54, 1.75)	1.48 (1.14, 1.66)
**1996**	5.05	1.85 (1.76, 1.95)	2.95 (2.60, 3.17)	3.13	1.61 (1.51, 1.72)	1.42 (1.09, 1.54)
**1997**	4.82	1.94 (1.84, 2.04)	2.95 (2.7, 3.26)	3.02	1.76 (1.65, 1.88)	1.50 (1.33, 1.70)
**1998**	4.64	1.96 (1.86, 2.06)	2.97 (2.71, 3.27)	2.98	1.80 (1.68, 1.92)	1.54 (1.34, 1.76)
**1999**	4.57	2.08 (1.97, 2.19)	3.12 (2.89, 3.39)	2.94	1.74 (1.63, 1.86)	1.40 (1.27, 1.63)
**2000**	4.37	2.07 (1.96, 2.18)	2.91 (2.69, 3.27)	2.87	1.72 (1.61, 1.84)	1.39 (1.22, 1.59)
**2001**	4.19	2.08 (1.97, 2.19)	2.85 (2.63, 3.15)	2.81	1.83 (1.71, 1.95)	1.45 (1.21, 1.66)
**2002**	4.12	2.16 (2.05, 2.28)	2.93 (2.74, 3.17)	2.76	1.91 (1.79, 2.04)	1.51 (1.36, 1.72)
**2003**	4.11	2.15 (2.04, 2.26)	2.93 (2.75, 3.24)	2.76	1.95 (1.83, 2.08)	1.67 (1.53, 1.87)
**2004**	3.93	2.03 (1.93, 2.14)	2.50 (2.33, 2.75)	2.62	1.84 (1.73, 1.97)	1.45 (1.24, 1.61)
**2005**	3.80	2.01 (1.90, 2.12)	2.57 (2.26, 2.73)	2.62	1.91 (1.79, 2.04)	1.94 (1.69, 2.18)
**2006**	3.69	2.05 (1.94, 2.16)	2.51 (2.24, 2.66)	2.50	1.77 (1.65, 1.89)	1.28 (1.11, 1.45)
**2007**	3.75	2.30 (2.19, 2.43)	2.84 (2.64, 3.10)	2.52	1.87 (1.75, 2.00)	1.37 (1.13, 1.52)
**2008**	3.65	2.32 (2.20, 2.45)	2.78 (2.61, 3.01)	2.49	1.99 (1.87, 2.12)	1.56 (1.41, 1.76)
**2009**	3.55	2.29 (2.17, 2.42)	2.76 (2.61, 3.03)	2.42	2.07 (1.94, 2.22)	1.71 (1.51, 1.82)
**2010**	3.46	2.27 (2.14, 2.39)	2.77 (2.55, 3.00)	2.36	2.09 (1.95, 2.23)	1.59 (1.49, 1.81)
**2011**	3.37	2.39 (2.27, 2.53)	2.85 (2.64, 3.00)	2.27	2.03 (1.89, 2.17)	1.46 (1.29, 1.61)
**2012**	3.30	2.40 (2.28, 2.53)	2.59 (2.47, 2.84)	2.23	2.11 (1.98, 2.25)	1.47 (1.32, 1.60)
**2013**	3.26	2.44 (2.32, 2.57)	2.81 (2.60, 2.95)	2.20	2.25 (2.11, 2.40)	1.73 (1.56, 1.86)
**2014**	3.26	2.39 (2.27, 2.51)	2.68 (2.48, 2.89)	2.21	2.40 (2.26, 2.56)	1.82 (1.65, 1.93)
**2015**	3.15	2.57 (2.44, 2.70)	2.77 (2.66, 3.04)	2.22	2.47 (2.32, 2.62)	1.78 (1.66, 1.97)
**2016**	3.21	2.49 (2.38, 2.62)	2.86 (2.69, 2.99)	2.23	2.27 (2.14, 2.42)	1.61 (1.51, 1.83)
**2017**	3.17	2.53 (2.40, 2.65)	2.67 (2.56, 2.93)	2.12	2.33 (2.19, 2.47)	1.74 (1.54, 1.82)
**% change**	-41.9	40.6	-11.0	-35.5	49.4	25.2

S1 Figure. Age-adjusted absolute and relative inequalities in adult premature mortality (deaths ages 18 to 74), Ontario by sex, 1992 to 2017.

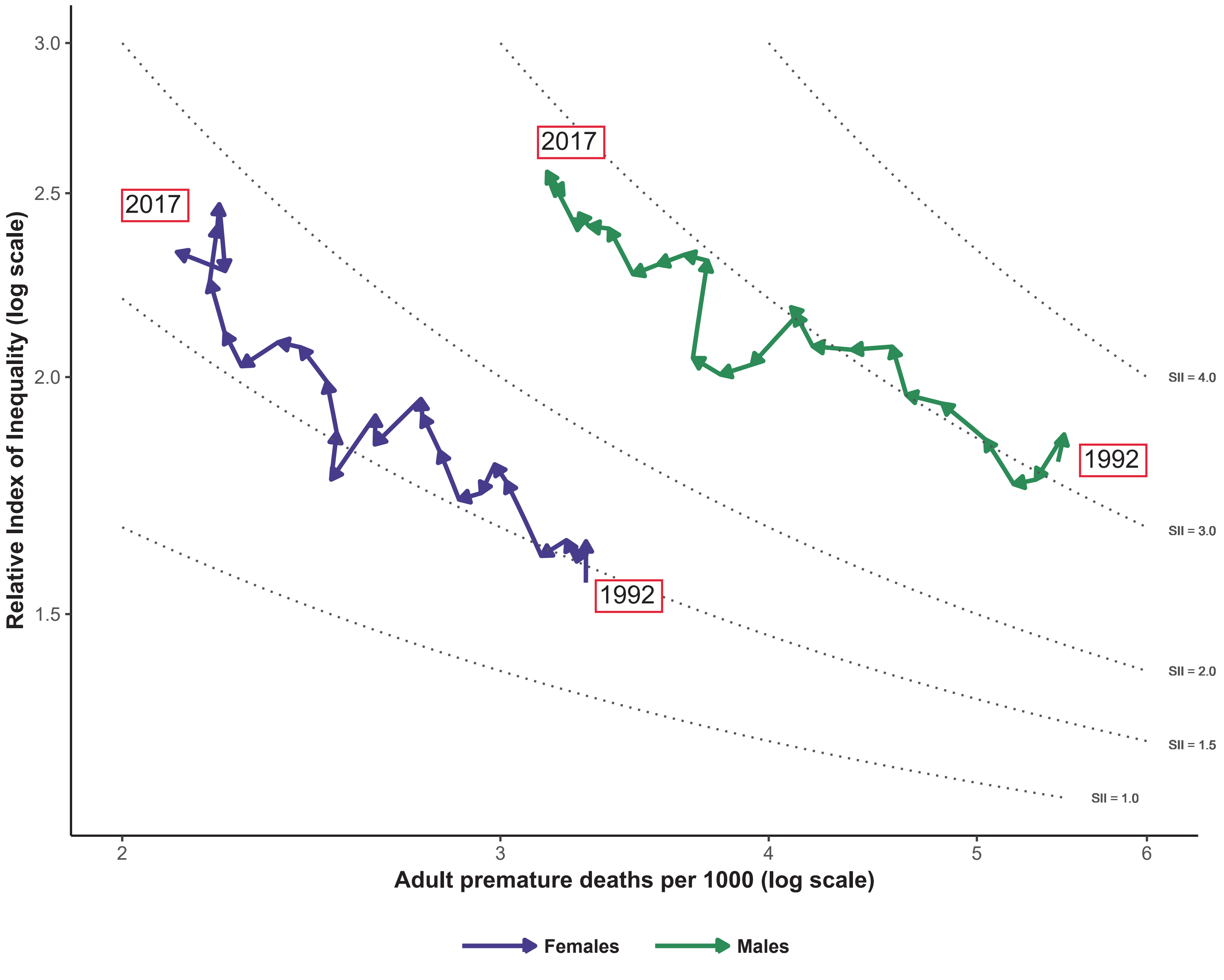

Supplement: S1 File — (PDF) [file pone.0230684.s001.pdf]
